# A Concept for Three-Dimensional Particle Metrology Based on Scanning Electron Microscopy and Structure-from-Motion Photogrammetry

**DOI:** 10.6028/jres.125.014

**Published:** 2020-04-29

**Authors:** Vipin N. Tondare

**Affiliations:** 1 Theiss Research, La Jolla, CA 92037, USA; 2 National Institute of Standards and Technology, Gaithersburg, MD 20899, USA

**Keywords:** algorithm variability, close-range photogrammetry, cylindrical substrate, maximum inscribed cylinder, mesh processing, multi-angle imaging, multiview geometry, point cloud, scale-invariant feature transform, size and shape, surface reconstruction, turntable method

## Abstract

Scanning electron microscopy (SEM) has been frequently used for size and shape measurements of particles. SEM images offer two-dimensional (2D) information about a particle’s lateral dimensions. Unfortunately, information about the particle’s three-dimensional (3D) size and shape remains unavailable. To resolve this issue, I propose a new concept in SEM: 3D particle metrology obtained by applying structure-from-motion (SfM) algorithms to multiple rotational SEM images of particles deposited onto a cylindrical substrate to generate a 3D model from which size and shape information can be extracted. Particles can have any size that is suitable for SEM imaging. SEM images of the sample can be acquired from 0° to 360° using a rotational-tip SEM substage. Here, I will discuss the concept and, for clarity, illustrate it with aquarium gravel particles that are glued onto a craft roll and imaged optically before generating the 3D model of that handmade craft. Future work will include the experimental SEM realization, as well as further development of the SfM algorithms. In my view, this proposed concept may become an integral part of SEM-based particle metrology.

## 1 Introduction

Particles are three-dimensional (3D) objects, and they mostly have nonspherical shapes [1, 2]. Different shapes of particles do exist [1, 3, 4]. Nanoparticles (1 to 100 nm size) are considered to be different than other particles because their properties significantly differ from bulk materials. They are used in many consumer goods such as clothes, cosmetics, drugs, foods, paints, sunscreens, varnishes, self-cleaning coating for floors, walls, and windows [5]. Sub-micrometer-size polystyrene latex particles are used for checking instrument calibration [6–8]. Metallic particles from hundred nanometers up to a few micrometers in size are used in the manufacturing of conventional electronic devices and crystalline silicon solar cells [9]. Metal particles with sizes typically ranging from a few micrometers to tens of micrometers are used in metal-based additive manufacturing [4]. In all these applications, particle size and shape primarily determine a particulate material’s performance. In the case of nanoparticle materials, size, shape, and size distribution are used for quality control as well as evaluating that material’s risk for people’s health and safety [5]. For instrument calibration and establishing measurement traceability, there is always a need for creating and maintaining particle size standards (reference materials) with a narrow size distribution [2, 10]. Consequently, the analysis of particle size and shape is routinely performed by industry, academics, government regulatory agencies, and nongovernmental organizations [11].

Measurement techniques such as dynamic light scattering, X-ray diffraction, small-angle X-ray scattering, laser diffraction, and single-particle inductively coupled plasma mass spectroscopy have limitations for the measurement of nonspherical particles, as their reported value is typically an equivalent spherical diameter [5, 12, 13]. On the other side, the atomic force microscopy (AFM) technique struggles with measuring lateral dimensions. It is known that the particle size and shape distribution analysis from scanning electron microscopy (SEM) or transmission electron microscopy (TEM) images are based on only two-dimensional (2D) information of lateral dimensions [5]. Additionally, during sample preparation for AFM, SEM, or TEM studies, nonspherical particles tend to settle with their largest side landing onto the flat substrate [5, 14, 15]. Obviously, the shape of these particles calculated from 2D information can be biased. In brief, the results from all these particle-sizing techniques differ, and, at present, no single technique gives reliable results of dimensional measurements. Usually, several techniques are used to characterize a parameter and minimize the measurement uncertainty [5, 16].

Laboratory-based X-ray computed tomography (XCT) is capable of determining the 3D size and shape of particles, but its spatial resolution cannot be as good as SEM for doing 3D nanometrology [4, 17]. In the literature, the concept of hybrid metrology [5] states that by using AFM and SEM techniques together, reliable dimensional measurements of nanoparticles can be done. However, in my view, it will be advantageous to develop a nondestructive method that uses only one instrument for the 3D measurements of particles having sizes ranging from nanoscale to tens of micrometers. Therefore, a particle measurement method based on SEM and structure-from-motion (SfM) photogrammetry is proposed.

### 1.1 SfM Photogrammetry

SfM photogrammetry [18] is sometimes also called “close-range photogrammetry” [19]. SfM photogrammetry has been used in geoscience, architecture, archaeology-paleontology, and engineering [18, 19, 20]. There exist many SfM photogrammetry software packages (hereinafter “SfM packages”), some of which are sold commercially [20]. The images for SfM photogrammetry can be captured either by moving the camera around the object, which is termed the “walk-around method” or by rotating the object within the field of view, termed the “turntable method” [20]. Multiple overlapping images of an object from different angles can have all the information that is necessary to create a 3D reconstruction [18]. The term “structure-from-motion” means the 3D structure is deduced from the relative motion of the camera [18, 19]. Instructions for capturing a set of good-quality images for SfM photogrammetry have been explained elsewhere [19, 20]. In general, the object should be well focused and positioned approximately in the middle of the image frame, and it should occupy most of the image frame. Multiple high-resolution, evenly illuminated overlapping images of the object are required to achieve a better quality of the 3D results.

The workflow [18] used in open-source SfM packages can be described very briefly as follows: First, an algorithm called the “scale-invariant feature transform” identifies distinctive invariant feature points across the set of overlapping images. Next, algorithms called “approximate nearest neighbor” and “random sample consensus” are used for matching the feature points across that image set. Next, the bundle adjustment package is used to produce a sparse point cloud. Next, a dense point cloud can be obtained by implementing algorithms called “clustering views for multi-view stereo” and “patch-based multi-view stereo.” After that, a mesh is built by connecting the points in the point cloud to represent the surface of the object. In the final step, texture mapping is done by using the 3D reconstruction and the source images to form a photorealistic 3D model of the original object.

### 1.2 SEM Imaging for SfM Photogrammetry

The SEM technique [21] is used for imaging and measuring objects with various dimensions ranging from millimeters to nanometers. SEMs offer high-contrast imaging with a high depth of field and excellent spatial resolution [21]. Additionally, SEM images of an object can be acquired from different viewpoints. In 2015, Eulitz and Reiss demonstrated an SfM photogrammetry–based 3D reconstruction of a rabbit kidney glomerulus using SEM images [22]. It was shown that SfM packages, which are made for processing optical images, are also able to process grayscale SEM images without “exchangeable image file” data [22].

**Fig. 1:**
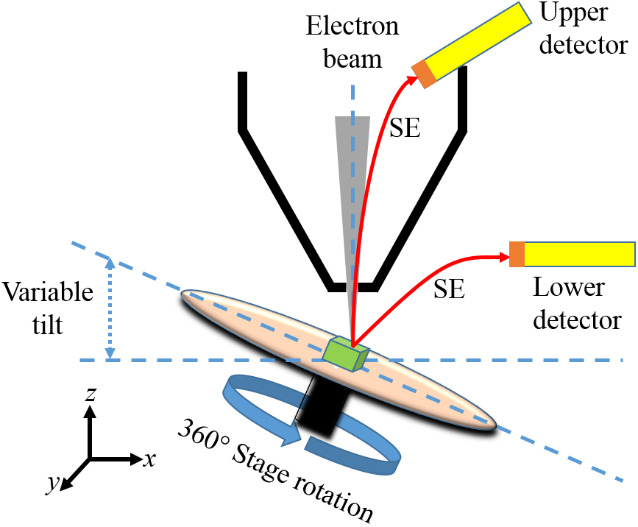
Schematic (not to scale) of multi-angle imaging method inside SEM.

It may be clear from the Sec. 1.1 that SfM photogrammetry differs [18, 19, 20] from the stereophotogrammetry (or traditional photogrammetry) technique [23]. SfM photogrammetry needs neither input images with the same magnification nor information about the angles between images [20]. Instructions for acquiring a set of SEM images [22, 24, 25] are basically the same as mentioned in optical close-range photogrammetry [19, 20]. Figure 1 shows that ordinary SEM stages can be tilted as well as rotated. Therefore, at different tilt and rotational angle settings, SEM images can be taken. In this way, multiple overlapping SEM images of an object can be acquired [22, 24–26]. This is nothing but the above-mentioned turntable method. It seems that roughly 35 to 70 SEM images are sufficient for building a 3D model of a simple object [22, 24–26]. However, the basic idea is to avoid or at least minimize blind zones in the case of complex objects, as far as possible, by taking more images from all necessary perspectives. Images captured using secondary electron (SE) mode [22] or backscattered electron mode [24] can be used for SfM photogrammetry–based 3D reconstruction. The choice of detector and SEM settings (*e.g*., working distance, aperture settings, beam landing energy, and exposure dose) [22, 24, 25] can vary with material and the size of the object under study.

### 2 The Proposed Concept of 3D Particle Metrology

**Fig. 2:**
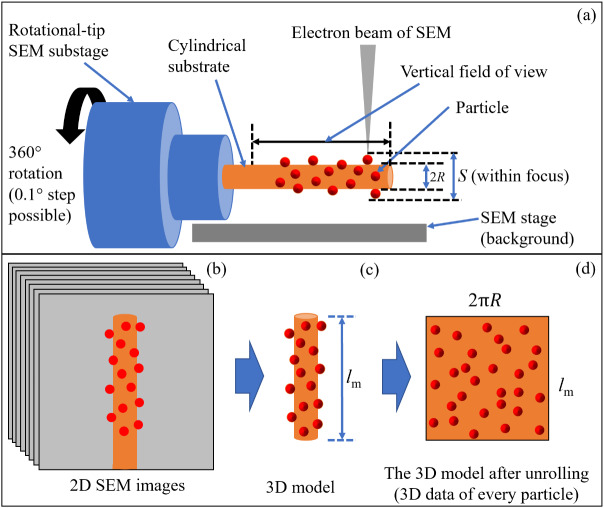
The proposed concept of 3D particle metrology based on SEM and SfM photogrammetry. This illustration is not to scale.

Figure 2 shows the proposed concept of 3D particle metrology. Figure 2(a) shows the method of acquiring images for 3D particle metrology using SEM. Although spherical particles are shown here deposited on the cylindrical substrate (hereinafter “CS”) of radius *R* for the simplicity of drawing, the particles can have any shape. The SEM sample, that is, the particles deposited on the CS, can be inserted into a rotational-tip SEM substage. For example, such a rotational-tip SEM substage^1^1Certain commercial equipment is identified here to adequately describe the experimental procedure. Such identification does not imply recommendation or endorsement by the National Institute of Standards and Technology, nor does it imply that the equipment identified is necessarily the best available for the purpose. can be capable of 360° rotation with 0.1° resolution [27]. Keeping the whole SEM sample (shown by the distance *S*) in focus, SEM images can be acquired at various rotational angles. Figure 2(b) shows a set of SEM images acquired at various rotational angles. This set of images can also have images acquired at different magnification or

working distance settings. Figure 2(c) shows that these SEM images can be used for generating a 3D model using SfM photogrammetry. Thus, the generated 3D model of length *l*_m_ is rich with 3D information of the particles under study. Figure 2(d) shows that a suitable mathematical computation program can be used to process the data, and finally extract the 3D information. The 3D information of every particle is then available for determining size and shape parameters.

Here, it should be noted that by using an SfM package, the sample’s surface texture in the form of 2D patches can be stored as a separate 2D image. Using such a 2D image (referred to as a UV map), a research group [25] has reported measurement of the size distribution of nanoparticles. However, in my view, the proposed concept in this article (as illustrated in Fig. 2) is different, systematic, and has obvious advantages.

## 3 Experimental Results

**Fig. 3:**
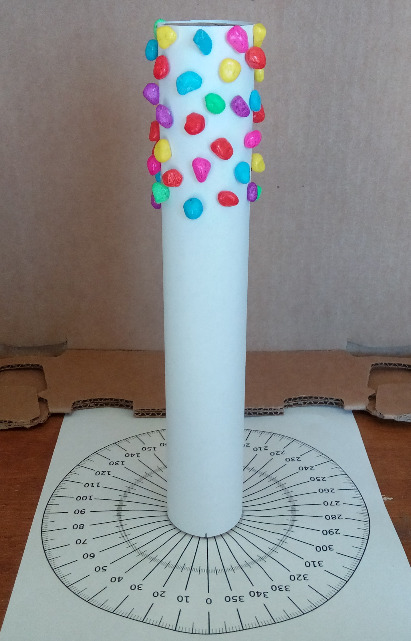
Experimental setup for representing the imaging method shown in Fig. 2(a).

To explain the proposed concept of 3D particle metrology in more detail, an SfM photogrammetry–based 3D reconstruction experiment using optical images of a handmade craft was performed. Figure 3 shows the experimental setup. Nonspherical-shaped aquarium gravel particles a few millimeters in size were used in this study. A craft roll covered with A4 size white paper was used as the CS. Aquarium gravel particles were glued onto that paper-covered craft roll, representing the SEM sample as shown in Fig. 2(a). This handmade craft was placed (see Fig. 3) onto and at the center of a printed copy of a 360° protractor. A fixed cellphone camera was used to take photos (*i.e.*, optical images) while manually rotating the handmade craft at every 5° rotational-angle interval. So, this experimental setup was representing the turntable-style SEM imaging method as proposed in Fig. 2(a). Photography conditions were less than optimum, which means neither a monochrome background curtain nor proper lighting was used.

**Fig. 4:**
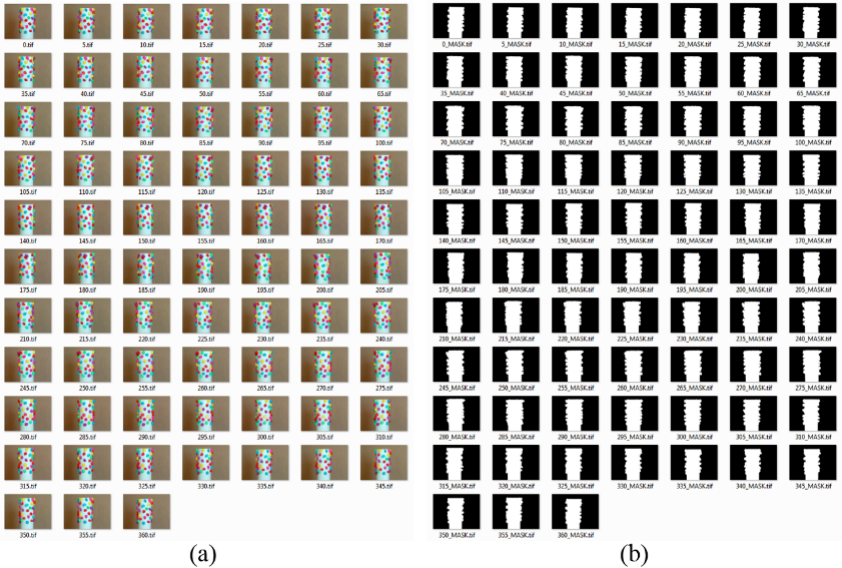
(a) Photos of the handmade craft, captured at every 5° rotational-angle interval and (b) the corresponding masks.

Figure 4(a) shows a collage of the 73 photos captured from 0° to 360° rotational-angle perspectives (*i.e*., a photo at every 5° interval). The photos taken at 0° and 360° rotational angles were basically the same. A commercially available SfM package was used to perform the 3D reconstruction. For the turntable SfM photogrammetry method, masking the background of the object is necessary [20]. It can also shorten processing time, because the SfM package will need to deal with relatively small areas inside the image frames. Further, accurate masking or accurate detection of the boundary of the object is necessary for an image-based dimensional measurement. Figure 4(b) shows the corresponding masks for all the images shown in Fig. 4(a). The SfM package used for the 3D reconstruction is also capable of masking the images. However, these masks were made externally, using a separate image processing software package.

**Fig. 5:**
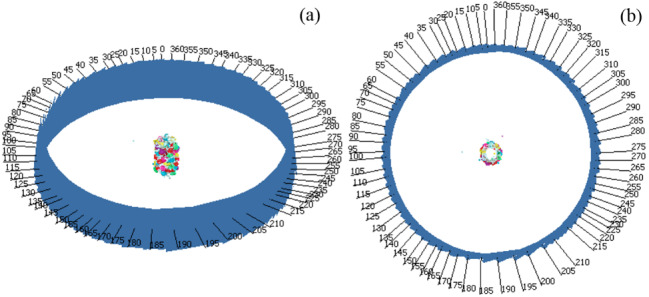
Sparse point-cloud model along with its camera positions: (a) tilted view and (b) top view.

Figure 5(a–b) shows the SfM package-generated sparse point-cloud model from a tilted view and the top view and the associated 73 camera positions. Figure 5(b) clearly shows that the SfM package identified the camera positions, and the unequal spacing in between the camera positions indicates that the handmade craft was not rotated at exactly a 5° interval. However, the rotational-angle accuracy does not matter for SfM photogrammetry. In fact, it also does not matter if the handmade craft had moved slightly from its original position (*i.e*., from the center of the 360° protractor) while taking the 5° rotational-angle steps. For the SfM photogrammetry–based 3D reconstruction, what really matters is the high-resolution, uniformly illuminated images of the object, captured from all necessary perspectives. Note that this discussion is useful for addressing concerns, if those exist, before performing the SEM experiment as shown in Fig. 2(a).

**Fig. 6:**
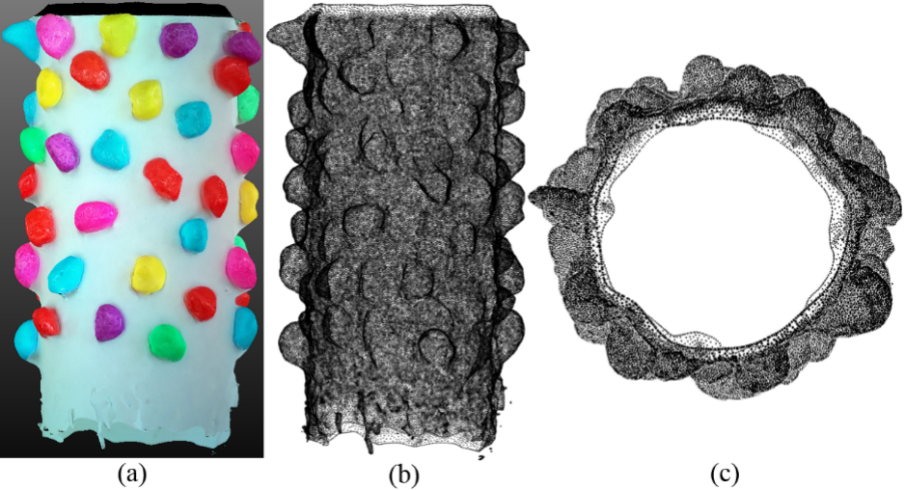
The SfM photogrammetry-generated 3D model, shown in the orthographic view mode: (a) front view, with the image texture, (b) front view in the mesh form, and (c) top view in the mesh form.

Figure 6(a) shows the SfM package-generated 3D model of the handmade craft, with the image texture added. Figure 6(b–c) shows a front view and the top view of the 3D model in the mesh form. Figure 6(c) shows that the craft roll used in this study was not a perfect cylinder; it is also likely that the CS used for preparing an SEM sample may not be a perfect cylinder. However, this issue can be taken care of mathematically by inserting a reference cylinder into the mesh model. This will be explained in Sec. 4.

**Fig. 7:**
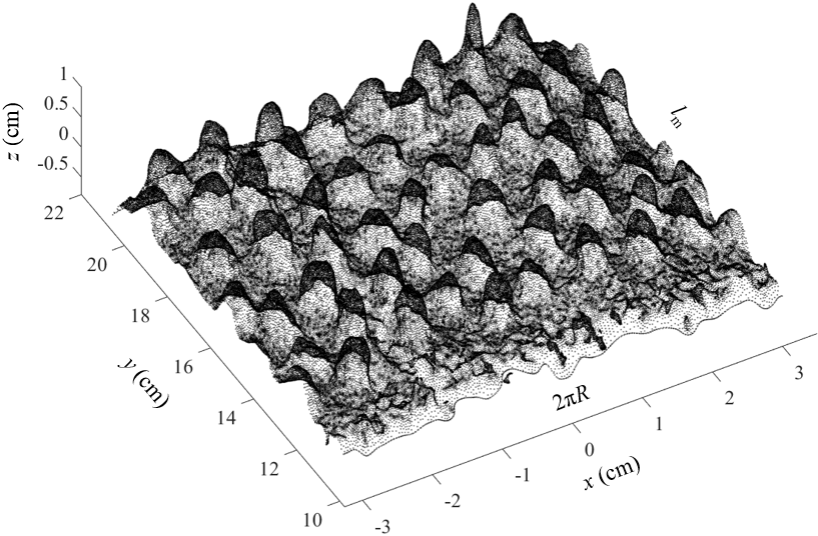
Unrolling of the cylinder-like mesh model. Here, the *z* axis represents the third dimension (height) of the particles.

Figure 7 shows that a cylinder-like mesh model, as seen in Fig. 6(b), can be unrolled using any suitable mathematical computation program. Note the 2π*R* shown in Fig. 7, was used to represent the circumference of the CS of the cylinder-like mesh model shown in Fig. 6(b), and the *l*_m_, was used to represent the length of the model. Now, in Fig. 7, the *z* axis represents the third dimension (height) of the particles.

## 4 Discussion

It should be noted that in the present study, although the experimental realization of the proposed concept using SEM images was not performed, as mentioned above, optical images or SEM images can be used for SfM photogrammetry–based 3D reconstruction. The proposed concept has been explained using an example of optical images of a craft roll with gravel particles. Figures 3 to 7 help to explain the proposed concept as described in Fig. 2. The only purpose of this exercise was to illustrate the point that a cylinder-like mesh model as seen in Fig. 6(b), generated by SfM photogrammetry, can be unrolled, and the 3D information about the particles can be extracted.

In my view, the SfM photogrammetry technique can certainly be used for 3D particle metrology, and the proposed concept can be optimized in further studies. Toward that goal, in this section, I will discuss a few relevant factors, for example, (Sec. 4.1) unobstructed viewing of particles, (Sec. 4.2) fabrication of the CS, (Sec. 4.3) particles with unobstructed views, (Sec. 4.4) number of particles measured per 3D model, (Sec. 4.5) load of particles on the CS, and (Sec. 4.6) SfM packages and measurement uncertainty.

### 4.1 Unobstructed Viewing of Particles

To achieve better 3D information for an object, it needs to be imaged from all necessary and experimentally possible viewpoints. For example, a single particle situated on a flat substrate can be imaged using a combination of various tilts and rotations of the SEM stage [25]. However, this setup is unsuitable for imaging many particles because there will not be unobstructed viewing of most of the particles, especially at high tilt angles. The aim of this article is to propose an efficient way of the 3D imaging of all particles of a particulate sample by SEM. In my view, particles deposited onto a CS is the best option. Here, the basic idea is to perform SEM imaging of each particle using a 180° unobstructed view.

The experiment with aquarium gravel particles (Fig. 3) was just for the purpose of demonstration. The particles were not glued sparsely on the craft roll. However, in the SEM experiment, the number of particles can be small enough on the CS for unobstructed viewing of each particle. It is known that particles can be deposited onto a substrate from liquid suspension or directly from the gas phase. In any case, preparation of the finest SEM samples would be extremely desirable. In this proposed concept, the focus is on particle sizes ranging from nanoscale to tens of micrometers. Can we place each particle onto the CS at desired locations, using the chemical template [28] or by some other method? It is certainly an interesting question for discussion, in the era of nanotechnology. In my view, SEM sample preparation methods for this proposed concept should be the topic of a separate study.

Once the SEM sample (*i.e.*, the particles deposited onto the CS) is ready, good-quality images, as mentioned previously, captured at every 5° rotational-angle interval, can be suitable for 3D reconstruction. As a rule of thumb, each image of the SEM sample should cover most of the area (at least around 70%) in the SEM image frame.

### 4.2 Fabrication of the CS

First, the diameter of the CS must be decided by considering the particle size under study. More about this will be discussed in Sec. 4.3 and Sec. 4.4. There are chemical or physical ways of fabricating the rod-like substrates. For example, the CS for the SEM study could be in the form of tungsten wire, and it could be easily prepared using dynamic electrochemical etching technique [29]. Alternatively, in Ref. [14], as required for their study, a carbon rod with a square cross section was fabricated using a focused ion beam.

For this proposed concept, the CS does not need to be a perfect cylinder, as demonstrated with the craft roll. However, during the fabrication process, it is desirable that the cylindricity tolerance (*C_t_*) of the CS should be kept as small as possible. The authors in Ref. [29] reported *C_t_* for the electrochemically etched tungsten wires below 10%. There could be other methods of fabrication of the CS, or other ways for achieving relatively better cylindricity. In any case, the surface of the CS may consist of a range of spatial frequencies [30]. The low-frequency components (referred to as “form”) may not be a matter of big concern. However, the amplitude parameters of the medium- and the high-frequency components (*i.e.*, of the “waviness” and “roughness,” respectively) should be kept well below the size of the smallest particle on the CS. Some roughness on the surface is not a problem, and it can be even useful for the feature points-matching in the SfM photogrammetry technique.

**Fig. 8:**
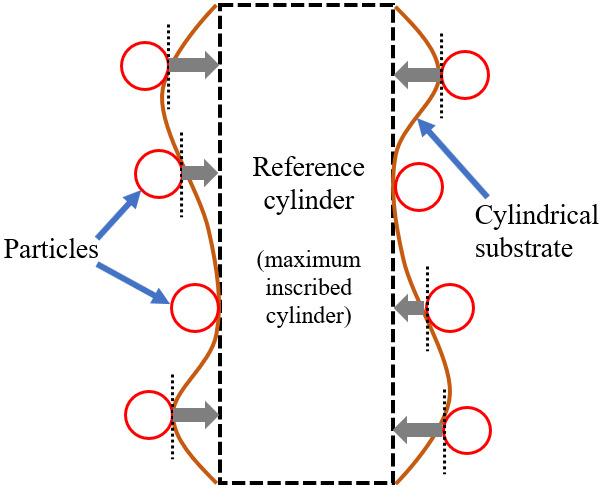
Schematic (not to scale) of the method to deal with the imperfect cylindrical substrate.

Although it was not done before producing Fig. 7, the 3D mesh model with its imperfect CS can be managed by fitting a maximum inscribed cylinder (MIC, also called a reference cylinder). Figure 8 illustrates the vertical cross section of the 3D mesh model with exaggerated imperfection of its CS. The black rectangle shows the vertical cross section of the MIC. Positions of the particles can be fixed on the reference cylinder mathematically.

### 4.3 Particles with Unobstructed Views

**Fig. 9:**
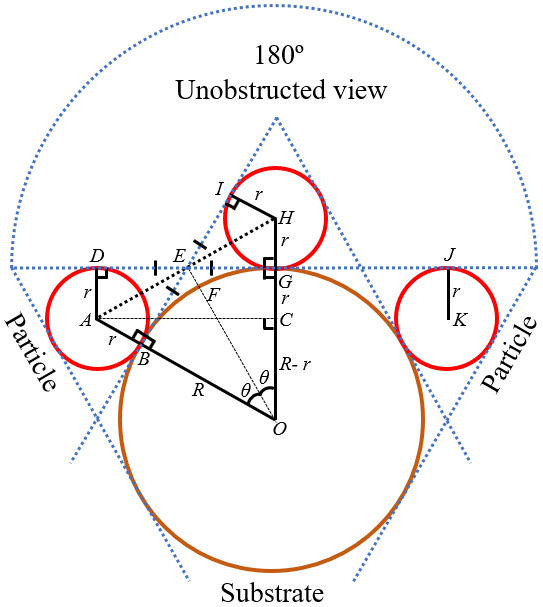
Schematic (not to scale) illustrating the criterion for an unobstructed view of a particle. Here, ‘*R*’ is the radius of the cylindrical substrate, ‘*r*’ is the radii of the particles, and ‘*θ*’ is the central angle. The arc length *BF* = the arc length *FG* = *R× θ*.

For estimating, the maximum number of particles with unobstructed views (*N*_max_) per 3D model, first, the criterion for an unobstructed view of a particle must be defined. Figure 9 is used for that purpose. It shows the schematic of the vertical cross section of the SEM sample from Fig. 2(a). The big circle with radius *R* is the CS, and the red-colored circles are the particles. All particles are considered to be of the same radius *r* for simplicity. Here, three particles are shown for an example. Each of these three particles can have a 180° unobstructed view for SEM imaging, because none of them can get hidden behind other particles. Thus, qualifying all of them as particles with unobstructed views.

In Fig. 9, the arc length *BG* that separates two particles depends on the radii of the CS and the particles. It is clear from Fig. 9 that, the maximum number of arcs (*Arcs*_max_) such as *BG* can be calculated as follows:

*Arcs*_max_ $=\frac{\pi }{\arctan\;(\sqrt{Rr}/R)}\;.$

Hence, the *N*_max_ on the circumference (*i.e.*, on a vertical cross section) of the CS is the number (*Nx*) calculated by rounding down the *Arcs*max from Eq. (1) to the nearest integer. Assume that the maximum acceptable number of particles on the considered height of the CS or on the length (*l*m) of the 3D model is the number (*Ny*) calculated by rounding down *l*m/4*r* to the nearest integer. So, the estimated *N*max per 3D model is equal to *Nx* × *Ny*.

The above estimation procedure certainly serves as a guideline for selecting the CS with a specific diameter and estimating the *N*_max_ per 3D model.

### 4.4 Number of Particles Measured per 3D Model

If the particles can be arranged artificially at desired locations on the CS, then the *N*_max_ per 3D model will be equal to *N_x_* × *N_y_*, as determined from Sec. 4.3. However, in this section, we will consider the more likely case that particles on the CS are deposited randomly. Now, a smaller number of particles present per 3D model will give better chance to have a higher percentage of particles with unobstructed views. Hence, a preliminary estimation will be necessary.

**Fig. 10:**
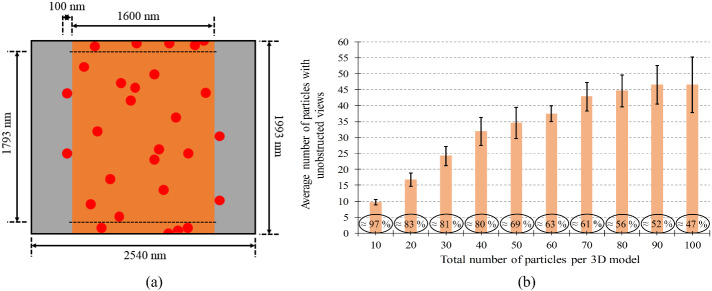
Estimating the number of particles with unobstructed views: (a) schematic (not to scale) of the 2D SEM micrograph, illustrating the considered dimensions, and (b) bar graph showing average number of particles with unobstructed views (and its percentage) for each case, and the error bars (black lines) showing 95% confidence intervals. The sample size, *n* = 6.

Figure 10 shows just an example, where the estimation work is done by considering 100 nm size particles. Figure 10(a) shows a schematic of the 2D SEM image of 100 nm size particles randomly deposited onto a CS. Here, it is considered that for SEM imaging of 100 nm size particles, a 2540 nm horizontal field width (HFW) of an SEM micrograph can be used. Further, the diameter of the CS is considered 1600 nm, so that the maximum width of the image of the SEM sample will be 1800 nm, which is ≈ 71% of 2540 nm. That means, as mentioned in Sec. 4.1, the image of the SEM sample covers around 70% of the image frame. Although the image of the CS can extend entirely along the height of the SEM micrograph, a 100 nm margin (*i.e*., equivalent to the considered particle size) from top and bottom has been left, and that part of the CS will not be considered in this estimation work. In this way, the final 3D cylinder-like mesh model can be thought of as having a CS of 1600 nm diameter and 1793 nm height (or, *l*m). The surface area of the CS will be ≈ 9 µm^2^, but there will be 850 full locations for 100 nm size particles, which can be obtained by considering the calculated circumference (5024 nm) and given height (1793 nm). The *N*_max_ per 3D model was estimated according to Sec. 4.3 and was found to be 96 particles. That means that even if the particles are deposited randomly, the upper limit of the allowed number of particles on the considered surface area is 96 particles. Now, the percentage of particles with unobstructed views per 3D model, from the total allowed ≤ 96 (say, < 100) particles per 3D model, needs to be found.

In a quick spreadsheet-based simulation, first, it was assumed that each particle has an equal chance to be on the CS at any location. However, it was considered that no more than one particle will occupy the same location. Obviously, some particles can happen to be adjacent to each other, and some can be relatively far from each other. Subsequently, random numbers with a uniform distribution were generated to represent locations of the considered number of particles per 3D model. The total number of particles with unobstructed views per 3D model was counted. Figure 10(b) shows the exemplary 10 cases of the total number of particles considered per 3D model. In Fig. 10(b), each bar represents the average number of particles with unobstructed views, calculated from the total of six trials generating random numbers. Since the sample size was small, *n* = 6, the *t*-distribution as a sampling distribution was considered, and the Student *t*-variate table [31] was used to calculate the upper and lower limit of 95% confidence interval (CI) in each case, as shown by the error bars (black lines).

These results indicate that, for example, if we allow (*i.e*., by preparing the SEM sample) 100 particles per 3D model, then we can say by considering the total population of such 3D models, an average 46.5 ± 8.6 (*P* = 95%) particles per 3D model will have unobstructed views, whereas if we allow only 10 particles per 3D model, then we can expect an average 9.7 ± 0.9 (*P* = 95%) particles per 3D model to have unobstructed views. Note that the 95% CI can be made relatively smaller, as required, by increasing the sample size. Basically, such an estimation work gives a hint for the upper limit of the particle number concentration to be maintained in the liquid suspension or the gas phase during the SEM sample preparation.

Here, it must be noted that although the length of the 3D model measured in this example is 1793 nm, the actual length of the particles-deposited CS can be more. That means another set of images can be acquired at an adjacent place without mounting another SEM sample inside the SEM. For future studies, it is also possible to envision a substage where more than one SEM sample can be mounted side by side. Automated image capturing would be certainly desirable in this case. In fact, in Ref. [24], for the first time, the authors showed the use of automated macros to run the SEM for stage movement and capture of images suitable for SfM photogrammetry–based 3D reconstruction.

The minimum number of particles that need to be measured, for an adequate representation of the population, also depends on the width of the particle size distribution. The International Organization for Standardization [32] gives a guideline regarding this issue. Using this guideline, it is possible to estimate the minimum number of particles that need to be measured for achieving a defined level of accuracy within a defined CI [32]]. This estimated number can be in the thousands [33] if the particulate material has a broad distribution of particle sizes, whereas this estimated number can be as small as 250 [5, 33] or even 100 [5, 34] if the particulate material has mostly one-sized particles (*i.e.*, a narrow particle size distribution).

### 4.5 Load of Particles on the CS

The distributed load of particles on the CS would depend on the choice of the particle material as well as the number and size of particles. The choice of the CS material may depend on the choice of the particle material as well as the method of the particle deposition. The CS can be made up of tungsten, iridium, silicon, carbon, or any other material compatible with electron microscopy.

For example, considering the dimensions of the CS of the 3D model as mentioned in the Sec 4.4 and its upper limit of the allowed number of 100 nm size particles, the mass of 96 gold particles as a percentage of the mass of tungsten-made CS would be only ≈ 1.4%, and 96 particles would not be necessary as concluded from Fig. 10(b). However, performing detailed nanomechanical calculations is beyond the scope of this article.

### 4.6 SfM Packages and Measurement Uncertainty

In this proposed concept of SfM photogrammetry–based 3D particle metrology, the faithful 3D reconstruction of the SEM sample would also depend on the quality of the SfM package. The best choice of the SfM package for a 3D reconstruction remains unsettled. The Appendix discusses this issue by comparing two SfM packages with the same input.

In order to estimate measurement uncertainty, two things are necessary: (1) simulated SEM images of a computer-generated virtual SEM sample having predefined dimensions and (2) an experimental sample with known particle sizes. For an experimental SEM study of the proposed concept, it may be noted that 100 nm size polystyrene spheres do exist (for example, National Institute of Standards and Technology [NIST] Standard Reference Material 1963a [35]). There exist metal particle size standards too [10]. The CS could be fabricated using the same technique as shown in Ref. [14], from an amorphous carbon layer. The CS can be even made up of silicon or other materials as possible, and a 1 nm thick osmium coating [36] on the SEM sample could be useful for achieving high-resolution SEM imaging. This is particularly required if the SEM sample is charged, or the signal-to-noise ratio is poor. It should be noted that the SEM study of this proposed concept needs to be done in a cleaned SEM chamber. Before this SEM study, the cleanliness of the SEM chamber should be inspected using the method developed by the NIST [37].

## 5 Conclusions

Particle size ranges from nanoscale to tens of micrometers are often used in various applications. There has always been a need for a nondestructive single method that can offer 3D size and shape information about every particle in a particulate sample. Although XCT is an excellent method for 3D size and shape analysis, its spatial resolution is not suitable for imaging nanoparticles, as compared to SEM. The SfM photogrammetry technique has been used for 3D reconstruction of an object using its SEM images. SfM photogrammetry demands neither images of the same magnifications nor information about their camera positions, which allows some tolerance in the accuracy of the SEM stages. However, I know of no published report describing SfM photogrammetry–based 3D reconstruction for more than one particle, as proposed here, intended for analyzing a particulate sample. The proposed concept of the CS for particle deposition is the best way to obtain unobstructed viewing of particles. A method for fabrication of a CS has been suggested, as well as a method for dealing with any imperfection that may remain during the manufacturing of the CS. For the analysis of a particulate sample, this article gives a clear guideline for choosing the diameter of the CS and maintaining a specific concentration of particle numbers during the SEM sample preparation. SEM sample preparation is challenging but seems doable using present technology. In future studies, this proposed concept can be demonstrated by SEM and then optimized. The estimation of the measurement uncertainty needs to be performed. The inconsistency of the SfM packages is certainly an issue. However, improvements in the algorithms and standardization of the SfM packages are inevitable, and, hence, the usefulness of this proposed concept for SEM-based particle metrology is assured. Further, in general, this proposed concept may lead to new developments in the image-based particle metrology.

## 6 Appendix

In general, commercially available SfM packages are “black box” in nature. Therefore, their accuracy remains unknown to the users. From a metrology perspective, then a legitimate question arises: Do different commercial SfM packages produce significantly different 3D reconstructions, given the same experimental data as input? This question becomes even more significant at a nanometer scale due to the required accuracy and precision in the measurements. In the present study, two different SfM packages (called Package *P-1* and Package *P-2*), which are commercially available and commonly used by researchers, were tested. The aim was to check the consistency of these SfM packages using the same experimental data.

**Fig. 11:**
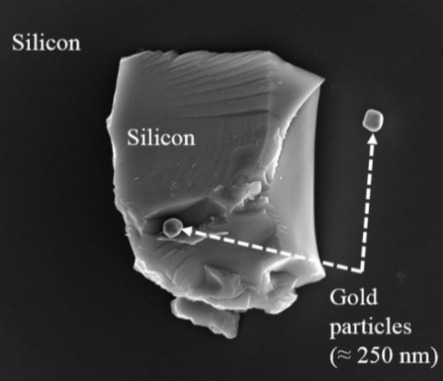
This silicon particle was imaged from 46 different perspectives using SEM. The horizontal field width is 6.35 µm.

For this study, gold particles (mean size ≈ 250 nm) were deposited onto a clean flat piece of the silicon wafer by a procedure described elsewhere [38]. A clean metal tweezer was used to scratch the surface of the gold particles-deposited silicon substrate. The resulting micrometer-scale silicon debris occasionally got mixed with gold particles. One such silicon particle, shown in Fig. 11, was selected for SEM imaging.

Before acquiring SEM images, the SEM chamber was cleaned using a low-power downstream plasma [37]. The experimental setup was as shown in Fig. 1. The upper (through-the-lens) detector was used in SE mode, for acquiring high-resolution and evenly illuminated SEM images of the silicon particle. SEM imaging parameters were as follows: beam voltage 5 kV, beam current 43 pA, beam dwell time 30 µs, and working distance 5 to 5.4 mm.

An SEM image at 0° stage tilt and 0° stage rotation and, a set of 45 SEM images from various perspectives, were acquired. A few tilt angles covering a range of −5° to 40° were used. Rotation steps were of 10°, 15°, or 20° as required to cover the object. Forty-four SEM micrographs each had an HFW of 6.35 µm, and two micrographs each had an HFW of 3.63 µm. The higher magnification images were taken to capture more details of the object. These 46 overlapped images reasonably (*i.e*., enough for this study) covered the object from various perspectives. These SEM micrographs were saved in the lossless TIFF file format. An image-processing software package was used to crop the micrographs to remove the information about image acquisition parameters usually present on SEM micrographs such as magnification, beam voltage, *etc*. All cropped micrographs were treated for optimum contrast and brightness. One of the SfM packages used in this study accepts only JPEG file format. Therefore, these files were exported at maximum quality in JPEG file format.

**Fig. 12:**
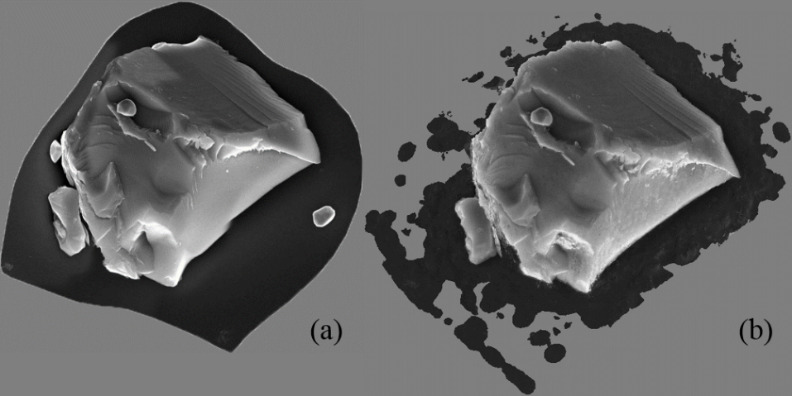
The 3D models produced by (a) package *P-1* and (b) package *P-2*.

These sets of JPEG files and TIFF files were input into Package *P-1* and Package *P-2*, respectively. In both cases, the package-recommended typical settings were used for generating 3D models. The workflow for these SfM packages is very similar. It generally involved loading images, aligning images, and building a sparse point cloud, building a dense point cloud, building a 3D mesh model, generating texture, and exporting results. The resulting 3D models can be viewed from any angle on the computer screen. Figure 12(a−b) shows the 3D models produced by Package *P-1* and *P-2*, respectively. Both 3D models are in orthographic view mode and viewed from the same perspective. Note that Package *P-1* tried to reconstruct the supporting silicon substrate including the gold particle, but Package *P-2* did not.

To compare these 3D models, first, it was decided to delete manually the parts of the mesh representing the silicon substrate. Package *P-1* and package *P-2* were used, respectively, to delete the silicon substrates of their 3D models before exporting those 3D models as Wavefront OBJ (object) files.

Unlike stereophotogrammetry, the 3D model generated by SfM photogrammetry lacks scale [18]. The 3D model by SfM photogrammetry is generated in a relative image-space coordinate system that needs to be aligned to a real-world object-space coordinate system by identifying a few features in a source image and corresponding orthographic view of the 3D model. The free software called “MeshLab” [39] was used to scale both 3D models by comparing them with the SEM image.

### 6.1 Comparison Results

The free software called “CloudCompare” [40] was used to compare these 3D models (in the mesh form). The metric called “Hausdorff distance” [41–44] was used to quantify the difference between these 3D models. For this purpose, the 3D model produced by Package *P-2* was used as the reference.

First, a coarse alignment of these 3D models was achieved. Following this step, the CloudCompare’s iterative closest point algorithm [40, 41] was used to fine-tune the alignment of these 3D models. After that, the CloudCompare used the vertices of the compared 3D mesh model and computed the distances for each of them relative to polygons of the reference 3D mesh model. These compared distances are signed, so that it becomes easy to identify the parts of the compared mesh that are inside and outside of the reference mesh. The resulting color scale associated with the compared mesh is then produced [40, 41–44].

**Fig. 13:**
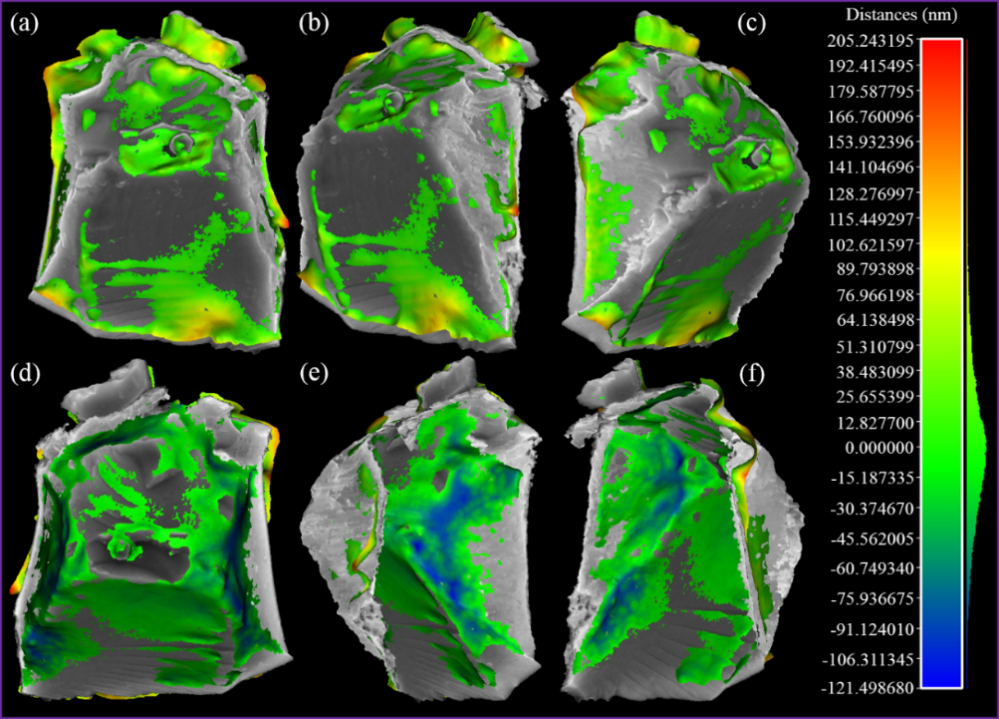

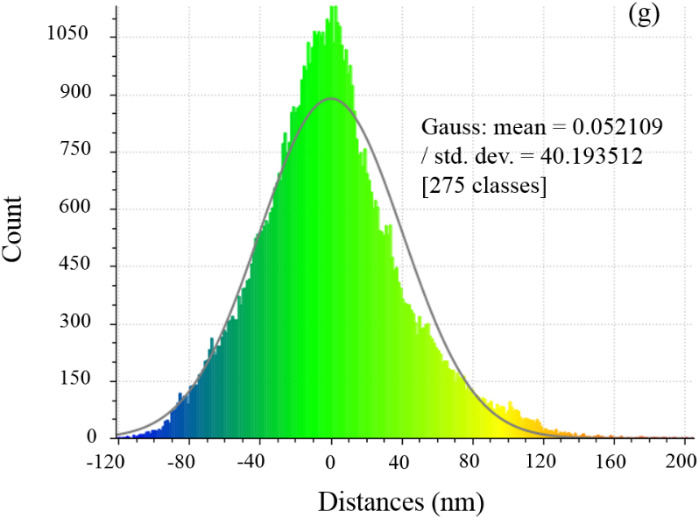
The differences between the 3D models generated by Package *P-1* (compared 3D model) and Package *P-2* (reference 3D model, gray color), shown in orthographic view mode: (a) top view, (b) top-right view, (c) top-left view, (d) backside view, (e) backside-right view, (f) backside-left view, and (g) the histogram plot of the signed distances.

Figure 13(a–f) shows the places where the 3D model generated by Package *P-1* differs from the 3D model (gray color) generated by Package *P-2*. The color scale (in nanometers) quantifies the differences. Here, a histogram, along with the color scale, illustrates the distribution of Hausdorff distances throughout the entire 3D model. The positive values indicate points of the 3D model from Package *P-1* that are above their matching point in the 3D model from Package *P-2*, and negative values indicate the reverse. The differences are ranging from 0.21 μm to −0.12 μm. Figure 13(g) shows the histogram plot of the signed distances. The best Gaussian fit has a mean of 0.1 nm and standard deviation of 40.2 nm.

It is clear that the two SfM packages gave slightly different results, and it remains unclear which (if any) SfM package produced the most accurate 3D model. There is still a need for a systematic study to understand whether the SEM images from a certain number of views can be stitched together using SfM photogrammetry with acceptable measurement uncertainty. Therefore, in future studies, first, the accuracy of the SfM packages may be determined by using simulated SEM images of a virtual SEM sample. True dimensions of a virtual SEM sample are known with mathematical accuracy, which is not possible in real SEM samples [45]. More details about using simulated SEM images for such a purpose can be found in earlier studies [45, 46].

SfM photogrammetry needs feature points matching in the available images [18]; however, it can be challenging at the nanometer scale. Moreover, the formal or complete definition of texture in the images remains unsettled [47]. However, Nurutdinova and Fitzgibbon have shown that 3D curves can be used to refine camera position estimation in challenging low-texture scenes [48], and Yezzi and Soatto have described an algorithm for the 3D reconstruction, under the assumption that the scene does not contain photometrically distinct “features” [49]. In brief, SfM photogrammetry remains a rapidly developing research field, and future improvements in algorithms are expected.
